# Low-Cost Control and Measurement Circuit for the Implementation of Single Element Heat Dissipation Soil Water Matric Potential Sensor Based on a SnSe_2_ Thermosensitive Resistor

**DOI:** 10.3390/s21041490

**Published:** 2021-02-21

**Authors:** Flávio Morais, Pedro Carvalhaes-Dias, Yu Zhang, Andreu Cabot, Fábio S. Flosi, Luis Caparroz Duarte, Adelson Dos Santos, José A. Siqueira Dias

**Affiliations:** 1 Faculty of Science and Engineering, Universidade Estadual Paulista (UNESP), Tupã, SP 17602-496, Brazil; flavio.morais@unesp.br; 2 Department of Electrical Engineering, Universidade Tecnológica Federal do Paraná (UTFPR), Cornélio Procópio, PR 86300-000, Brazil; pcdias@utfpr.edu.br (P.C.-D.); lfduarte@utfpr.edu.br (L.C.D.); 3 Catalonia Institute for Energy Research (IREC), Jardins de les Dones de Negre 1, 08930 Barcelona, Spain; peterzhang@irec.cat (Y.Z.); acabot@irec.cat (A.C.); 4 Catalan Institution for Research and Advanced Studies—ICREA, Pg. Lluís Companys 23, 08010 Barcelona, Spain; 5 Department of Semiconductors, Instrumentation and Photonics, School of Electrical and Computer Engineering, University of Campinas, Campinas, SP 13083-820, Brazil; fabioflosi@interair.com.br (F.S.F.); adelson@dsif.fee.unicamp.br (A.D.S.)

**Keywords:** heat dissipation matric water potential sensor, heat dissipation soil matric potential sensor, single-element soil matric water potential sensor, power control circuits, signal conditioning circuit

## Abstract

A low-cost signal processing circuit developed to measure and drive a heat dissipation soil matric potential sensor based on a single thermosensitive resistor is demonstrated. The ${\mathrm{SnSe}}_{2}$ has a high thermal coefficient, from $-2.4\;\Omega {/}^{{}^{\circ}}$C in the 20 to 25 ${}^{{}^{\circ}}$C to $-1.07\;\Omega {/}^{{}^{\circ}}$C in the 20 to 25 ${}^{{}^{\circ}}$C. The ${\mathrm{SnSe}}_{2}$ thermosensitive resistor is encapsulated with a porous gypsum block and is used as both the heating and temperature sensing element. To control the power dissipated on the thermosensitive resistor and keep it constant during the heat pulse, a mixed analogue/digital circuit is used. The developed control circuit is able to maintain the dissipated power at 327.98±0.3% mW when the resistor changes from 94.96Ω to 86.23Ω. When the gravimetric water content of the porous block changes from dry to saturated ($\theta_{w}=36.7\%$), we measured a variation of 4.77Ω in the thermosensitive resistor, which results in an end-point sensitivity of 130 mΩ/%. The developed system can easily meet the standard requirement of measuring the gravimetric soil water content with a resolution of approximately $\Delta \theta_{w}=1\%$, since the resistance is measured with a resolution of approximately 31μΩ, three orders of magnitude smaller than the sensitivity.

## 1. Introduction

When applied in large crop fields, advanced irrigation management systems used in precision agriculture [1] require low-cost, accurate and reliable soil matric potential sensors (or water content sensors), which can be done using several types of sensors, as capacitive probes [2], time-domain reflectometers [3], tensiometers [4] and heat dissipation sensors [5]. Due to the low-cost, small size and durability when inserted into the soil, heat dissipation soil water sensors are frequently employed in crop fields. There are basically two types of heat dissipation soil water sensors: dual heat pulse probe (DHPP) and single heat pulse probe (SHPP), and both are briefly discussed in the following sections.

### 1.1. DHPP Sensors

The DHPP is a sensor with two fixed probes: one contains a heater (usually a NiCr resistor) and the other has a temperature sensing device (usually a thermocouple or a thermistor). The two probes are separated by a small distance *r* (r≈6 mm), and after applying a well-controlled heat pulse to the heater probe (typically during 8 s), the maximum temperature reached in the other probe is measured. This maximum temperature depends on the soil thermal conductivity which, in turn, depends on its water content. After proper calibration, performed in the specific soil where it will be used, the maximum measured temperature can be correlated with the amount of water in the soil [6].

However, the probes of conventional DHPP are made with thin stainless steel needles that can deflect when inserted into the soil, resulting in large errors in the estimation of the soil water content. Liu et al. showed that a 11% error was measured when one of the probes suffered a $1^{{}^{\circ}}$ deflection, and this is a problem that is very difficult to overcome during installation of the sensor into the soil [7]. To eliminate this weakness, needle-less DHPP sensors have been developed [8,9].

### 1.2. Conventional SHPP Sensors

In the SHPP both the heating and the temperature sensing elements are inserted in the same probe [10]. With the probe inserted into the soil, a constant power source is applied to the heater and, after a period of 20 to 30 s, the temperature rise ΔT (or an electrical parameter that depends on temperature) is measured. The thermal conductivity of the soil depends on the amount of water absorbed in it, and since the soil acts as a heat sink to the probe, the soil moisture can be estimated using this technique.

### 1.3. Sensors Encapsulated in Porous Blocks

Both DHPP and SHPP require a good contact with the soil, because if the contact between the soil and the probes is not perfect, the thermal conductivity of the soil will not be measured properly. To solve this problem, the probe is encapsulated with a porous block, producing a heat dissipation soil matric potential sensor.

When a porous block is inserted into the soil, the water flows between the soil and the porous element (in both directions) until an equilibrium is reached. In this case, the SHPP sensor measures the thermal conductivity of the ceramic/soil complex, and a correlation between the thermal conductivity and the matric potential of the soil can be determined. Soil water potential sensors are commonly employed in precision agriculture irrigation management, and many commercial systems are available [11,12].

### 1.4. Single-Element SHPP Sensors

Recently, Dias et al. presented a novel type of SHPP soil water content sensor, where a single element (an NPN transistor) performed as both heating and sensing elements [13]. In the first version of the sensor, the transistor was inserted directly into the soil, while in a recent and more sophisticated version, the transistor was encapsulated with a porous ceramic block [14]. The bipolar transistors present a temperature variation of the base-to-emitter voltage $V_{BE}$ of approximately $dV_{BE}/dT\approx -2\;\mathrm{mV}{/}^{{}^{\circ}}$C. However, to detect soil water content with high resolution, a sensor with a heating/measuring element that presents a higher thermal coefficient (TC) is desired.

In this paper we present a single-element SHPP based on a thermosensitive resistor fabricated using nanocrystalised materials, that acts as both heating and temperature sensing element. The thermosensitive element changes its resistance as power is applied to it (due to its temperature increase) and to maintain the power dissipated on it constant (as required by the SHPP technique), we developed a simple and low-cost mixed circuit, based on a microcontroller MSP430AFE232 (from Texas Instruments, Dallas, TX, USA).

## 2. Principle of Operation of a SHPP Sensor

The conventional fabrication technique of SHPP soil water potential sensors with porous material consists of encapsulating two elements, (one heating and one temperature sensing element) with a porous block [11,12]. In our sensor we used a single element, a thermosensitive resistor, encapsulated with a porous gypsum block.

The variations of the thermal conductivity of a porous material depends on the amount of water present in the porous body. The temperature rise caused by the heat exchange between the heater element and the porous body is measured after the end of the application of a heat pulse of fixed duration. For a line-heat source buried in an infinite medium, with an initial temperature $T_{0}$, when a heat pulse of duration $t_{f}-t_{0}$ is applied to the heating element, the temperature change ΔT measured in the sensing element at the end of this pulse was approximated by [15] as:

(1) $$\Delta T=T_{f}-T_{0}=-\frac{q}{4\pi k}ln\left(t_{f}-t_{0}\right)$$

where *k* is the thermal conductivity of the medium ($W\;m^{-1}{\;}^{{}^{\circ}}$C${}^{-1}$), *q* is the heat input per length of the heater ($W\cdot m^{-1}$), $T_{0}$ is the initial temperature (measured at time $t_{0}$), and $T_{f}$ is the final temperature (measured at time $t_{f}$). Solving for *k* in Equation (1) we can write:

(2) $$k=-\frac{q}{4\pi \Delta T}ln\left(t_{f}-t_{0}\right)$$

Therefore, using the measured value of ΔT, it is possible to estimate the value of the thermal conductivity *k* of the porous material moistened with water and measuring the value of ΔT, it is possible to correlate the value of ΔT with the amount of water in the porous block.

## 3. Temperature Sensor Using Thermosensitive Resistors

When using SHPP heat dissipation soil water potential sensors in agriculture, the measured values of the ΔT are generally low, typically in the order of 3 ${}^{{}^{\circ}}$C [11]. Thus, a temperature measurement technique with high accuracy and resolution better than 0.1 ${}^{{}^{\circ}}$C is usually required, and in conventional SHPP sensors with NiCr resistors and a type K thermocouples, we need to measure voltages in the order of 4 μV.

Therefore, to simplify the design of the signal processing circuits, a sensor with high thermal sensitivity is desirable. Several strategies can be used to increase the thermal sensitivity of a bulk material [16]. These include the incorporation of a high density of electroactive defects that are activated in the temperature range of interest and the introduction of a high density of potential barriers through nanostructuration of the material. We selected SnSe2 as thermosensitive material because it intrinsically contains a large density of electroactive selenium vacancies and it is characterized by a 2D structure that, when processed in solution at moderate temperatures, results in a large density of grain boundaries. We combined SnSe2 with ZnO to adjust the overall resistance of the composite to a proper range that facilitated the signal processing. The process of fabrication of the thermosensitive resistor is described next.

### Thermosensitive Resistor Based on Nanocrystalized Materials

${\mathrm{SnSe}}_{2}$ nanoparticles were produced by thermal decomposition at $380{\;}^{{}^{\circ}}$C of a Sn-Se precursor solution prepared by dissolving 10 mM Sn powder (Sn, ≥99.0%, from Sigma-Aldrich, St. Louis, MO, USA) and 20 mM Se powder (Se, 200 mesh 99.5%, from Acros Organics, Geel, Belgium) within 20 mL of oleylamine ($C_{18}H_{35}{\mathrm{NH}}_{2}$, ≥98%, from Sigma-Aldrich) and 0.2 mL ethanedithiol ($C_{2}H_{4}{\left(\mathrm{SH}\right)}_{2}$), ≥98.0%, from Sigma-Aldrich). Nanomaterials obtained from the consolidation of the produced ${\mathrm{SnSe}}_{2}$ nanoparticles were characterized by very high electrical conductivities ($100\;Sm^{-1}$) and a very abrupt temperature dependence in the temperature range around ambient [17]. To adjust the electrical resistance of the resistor within a proper range (200–500 Ω), the high electrical conductivity ${\mathrm{SnSe}}_{2}$ nanopowder was mixed with a commercial ZnO powder (ZnO, 99%, from Sigma-Aldrich) at a weight ratio $m_{{\mathrm{SnSe}}_{2}}:m_{\mathrm{ZnO}}=7:3$. This mixture was hot-pressed at $400{\;}^{{}^{\circ}}$C and 60 MPa for 3 min to produce the thermosensitive resistor. The final thermosensitive resistor $R_{T}$ was a small cylinder (diameter D=4 mm and length L=8 mm), with contacts made with an electrically conductive silver epoxy adhesive (AA-DUCT 902-2.5, from Atom Adhesives, Providence, RI, USA).

## 4. Signal Conditioning Circuit

**Figure 1:**
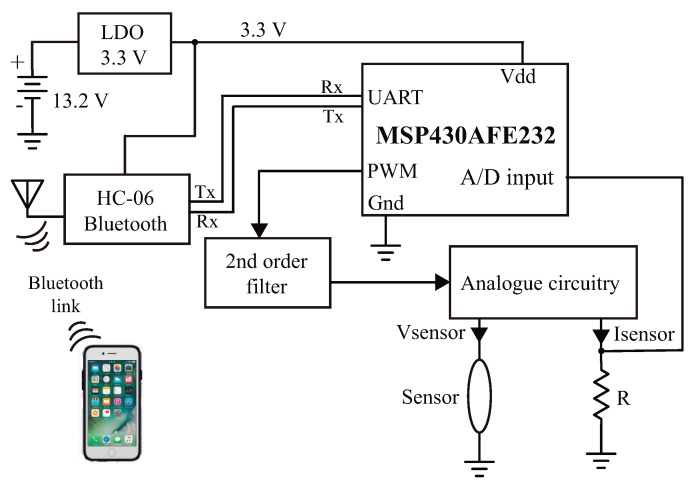
Block diagram of the sensor signal processing circuit.

When a soil water sensor uses a single device as both heating and temperature sensing element, especial techniques must be used to apply a constant power to the device (as presented in [18], where a transistor is used as the sensor). In this work we use a mixed signal circuit to control the power applied to the thermosensitive resistor and measure its temperature variation. A block diagram of the circuit is shown in Figure 1.

### 4.1. Power Supplies and Digital Circuits

The system is powered with 2 lithium-thionyl chloride LS 2500 batteries (from Saft, Levallois-Perret, France) in series, generating a power supply $V_{cc}=7.2$ V. A 3.3 V LDO (low-dropout) voltage regulator TPS70933 (from Texas Instruments) was used to power the microcontroller and the Bluetooth module (HC-06, from Guangzhou HC Information Technology Co., Guangzhou, China).

The MSP430AFE232 microcontroller (from Texas Instruments) is configured to operate with a master clock of $f_{ck}=12$ MHz. It manages the entire signal conditioning circuit, performing several actions: it runs the power control algorithm, calculates several parameters ($R_{T}$, temperature, $V_{sensor}$, the value of the Pulse Width Modulation variable (pwm), transmits the data via its UART interface, reads the voltage $V_{\left(R_{6}\right)}$ with a 15 bit unipolar analog-to-digital (A/D) converter, and provides a PWM output signal using Timer 1, with a frequency of 12 kHz.

### 4.2. Analogue Section

**Figure 2:**
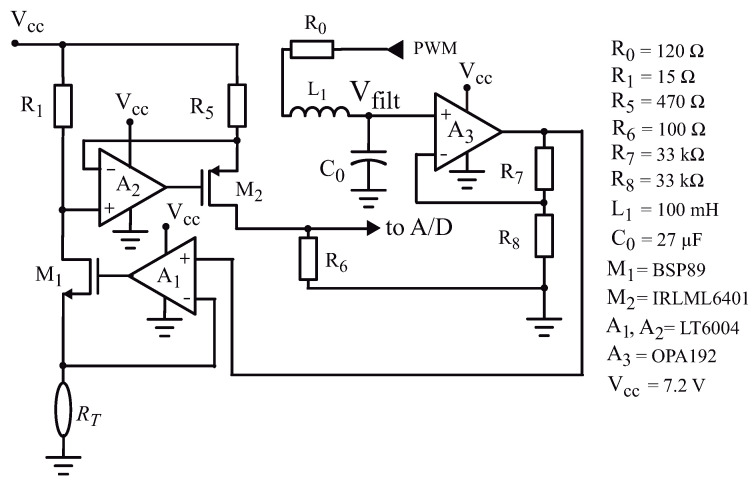
Schematic diagram of the sensor analogue signal processing circuit.

The analogue section of the circuit is presented in Figure 2. The PWM output of the microcontroller is sent to a second order RLC filter, and the output of the filter is amplified (with a gain $G_{v}=1+R_{7}/R_{8}=2$) by op-amp A3. This amplification is necessary because the maximum output voltage of the filtered PWM is 3.3 V (the supply voltage $V_{dd}$ used to power the microcontroller), and with 3.3 V we would be limited to apply only ≈135 mW to the sensor. Using A3 (a rail-to-rail op-amp, OPA192 from Texas Instruments), with the gain $G_{v}=2$ we can apply up to 6.6 V to the sensor, and with this voltage we can apply a heat pulse of P≈550 mW.

Op-amp A1 (LT6004, from Analog Devices, Wilmington, MA, USA) is connected as a follower, with the NMOS current buffer M1 inside the feedback loop. Neglecting the off-set voltage and the input bias current of A1, we can say that the output voltage of A3 is applied on $R_{T}$.

The current on $R_{T}$ ($I(R_{T})$) is furnished by the source of M1. The drain and the source currents in a MOS transistor are equal, so that $I(R_{T})$ flows through resistor $R_{1}$. The voltage on $R_{1}$ ($V_{R_{1}}=R_{1}I\left(R_{T}\right))$ is transferred by op-amp A2 (LT6004) to resistor $R_{5}$, generating a current in $R_{5}$ given by $I\left(R_{5}\right)=V_{R_{1}}/R_{5}$. This current goes into the source of the PMOS transistor $M_{2}$ and, when it leaves its drain, develops a voltage on resistor $R_{6}$ given by $V_{R_{6}}=I\left(R_{5}\right)R_{6}$. Thus, we can write that:

(3) $$I\left(R_{T}\right)=\frac{V_{R_{6}}}{R_{6}}\frac{R_{5}}{R_{1}}$$

**Figure 3:**
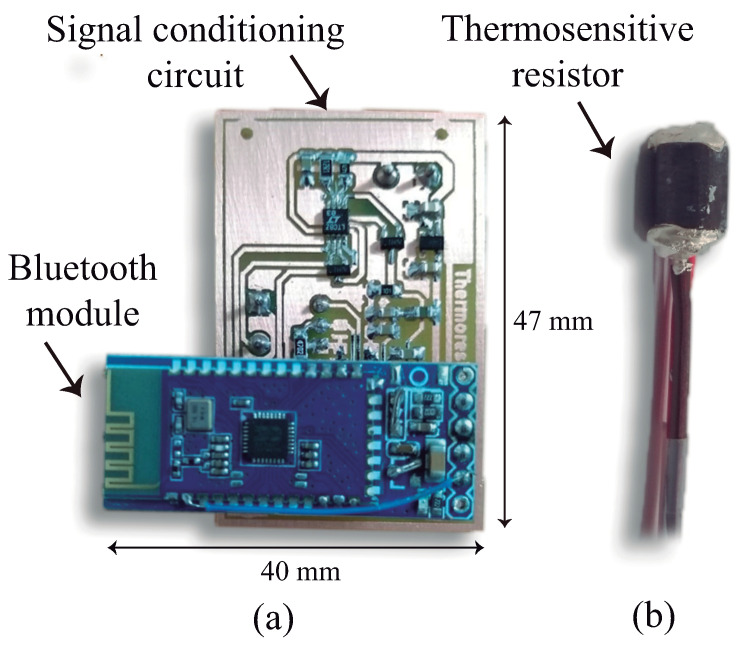
Photograph of the: (**a**) PCB of the signal processing circuit with the bluetooth module; (**b**) ${\mathrm{SnSe}}_{2}$ cylinder used as the thermosensitive element of the sensor.

A photograph of the ${\mathrm{SnSe}}_{2}$ thermosensitive resistor and the double layer PCB is shown in Figure 3.

## 5. Results

### 5.1. Characterization of the PWM

Since the filtered PWM voltage is used to apply the power to the thermosensitive resistor, it is necessary to characterize precisely the voltage $V_{sensor}$ as a function of the PWM. After the signal processing circuit is assembled, we inserted a resistor (*R* = 1 kΩ) in the contacts of the sensor, and a special program, that accepts inputs of PWM values via the Bluetooth connection with a smartphone, is loaded in the microcontroller.

**Figure 4:**
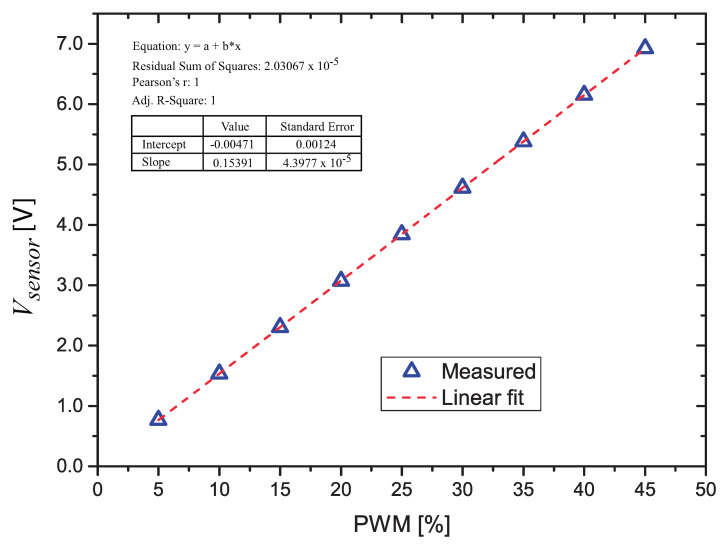
Measured voltage across $R_{T}$ as a function of the Pulse Width Modulation (PWM) value.

The measured values are plotted and the linear fitting of these points (calculated using a simple linear regression technique) is calculated (Figure 4). The resulting equation (Equation (4)) is used in the final microcontroller firmware, so it is possible to assign PWM values to obtain a given voltage on the sensor. It is important to notice that by measuring the voltage directly on the sensor’s terminals we eliminate the errors due to the off-set voltage and input bias current in op-amps A1, A3 and in the gain of A3, that depends on $R_{7}/R_{8}$ relation.

We performed the characterization of the PWM with values from 5.0% to 45.0%. The voltage on the *R* = 1 kΩ resistor as a function of the PWM value was measured with a HP 3468A multimeter.

(4) $$V_{sensor}=0.15391\cdot \left(pwm\right)-0.00471\;\left[V\right]$$

**Figure 5:**
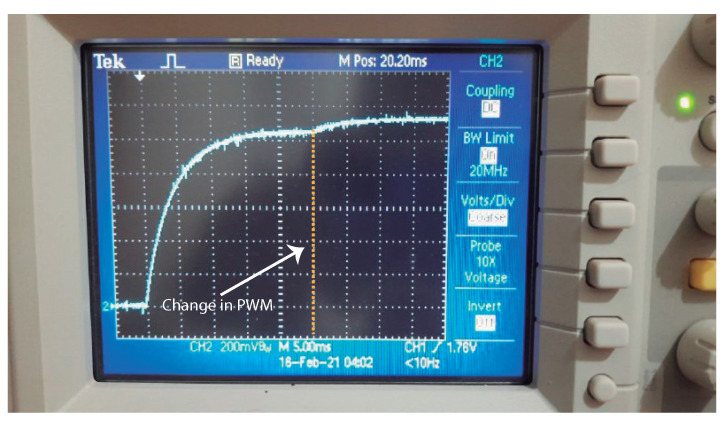
Transient response of the filter.

In the next sections we will show that, during the operation of the system, the value of $V_{sensor}$ changes only within the range $4.83\leq V_{sensor}\leq 5.17$, which corresponds to 31.4≤PWM≤33.6. Since $V_{sensor}$ is applied using the second order filter shown in Figure 2 ($R_{0},L_{1},C_{0}$), we measured the transient response of the filter starting from zero and then using a change from the minimum to the maximum values of the PWM. The measured result is shown in Figure 5. The filter was calculated to provide a critically damped response (with $\sqrt{L_{1}C_{0}}=2L_{1}/R_{0}$. As we can see, at the start of the PWM signal we have a small delay (approximately 18 ms) before the voltage $V_{filt}$ reaches the steady state. We changed abruptly the PWM from 31.4% to 33.6% at t=25 ms, and it is possible to notice that the value of $V_{filt}$ reaches the new steady state in approximately 10 ms.

As we can observe, the settling time of the filter (both at the start of operation and after the PWM is changed between two extreme values) is less than 18 ms and 10 ms, respectively, which is much smaller than the period between two A/D conversions, 250 ms.

Since the PWM has to be defined as an integer, we used the PWM variable (pwm) changing from 0 to 1000, corresponding to a PWM value of 0% to 100%, and this results in a resolution of 0.1% in the PWM’s value. Using pwm changing from 0 to 1000 we, consequently, defined the PWM frequency as $f_{PWM}=12$ kHz, since it is given by $f_{PWM}=f_{clk}/1000$. Therefore, for a variation ΔPWM=0.1, using Equation (4) we can calculate the variation $\Delta V_{sensor}=1.54$ mV. If the voltage in the sensor changes from $V_{0}$ to $\left(V_{0}+\Delta V_{sensor}\right)$ we can estimate the change in the power dissipated in the thermosensitive resistor as:

(5) $$\Delta P_{min}=\frac{2V_{0}\Delta V_{sensor}+{\left(\Delta V_{sensor}\right)}^{2}}{R_{T}}$$

Thus, if the sensor resistance is $R_{T}=80\;\Omega$ ($T=22{\;}^{{}^{\circ}}$C) and we apply a heating power P=300 mW to it (with $V_{sensor}=4.9$ V), we obtain from Equation (5), $\Delta P_{min}\approx 0.19$ mW, approximately 300mW±0.06%. Of course this is just an estimation, since in Equation (4) we considered that $R_{T}$ was constant.

### 5.2. Characterization of the Thermosensitive Resistor

The first experiment was the characterization of the thermosensitive resistor, using a very small (10 cm × 10 cm × 10 cm) thermoelectric environmental chamber. We conducted the characterization in the 20–40 ${}^{{}^{\circ}}$C range to obtain more information about the ${\mathrm{SnSe}}_{2}$ material. The value of the resistor was measured with a 3468A 5.5-Digit Digital Multimeter from HP, using a 4-wire method.

**Figure 6:**
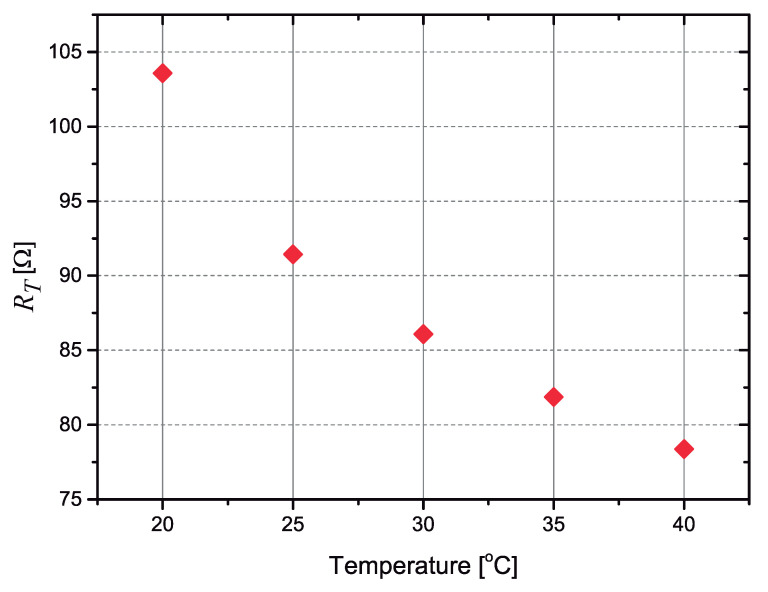
Measured values of the resistance as a function of the temperature for the ${\mathrm{SnSe}}_{2}$ resistor.

A plot of the measured resistance as a function of temperature is shown in Figure 6. The resistor presents a $TC\approx -1.5\%{/}^{{}^{\circ}}$C, corresponding to a variation $\Delta R_{T}/\Delta T\approx -1.8\;\Omega {/}^{{}^{\circ}}$C). If 50 mA is passed through this resistor, a voltage variation of $\Delta V_{R_{T}}\approx -90\;\mathrm{mV}{/}^{{}^{\circ}}$C is obtained, while in [13] the bipolar transistor used to measure the temperature presents a variation of the base-to-emitter voltage that is much smaller, typically −1.8 to −2.2 mV/${}^{{}^{\circ}}$C.

We used the points at $T=20{\;}^{{}^{\circ}}$C, $T=30{\;}^{{}^{\circ}}$C and $T=40{\;}^{{}^{\circ}}$C (from the measured data presented in Figure 6) to calculate the coefficients of the Steinhart–Hart equation for the ${\mathrm{SnSe}}_{2}$ resistor:

(6) $$\frac{1}{T}=A+Bln\left(R_{T}\right)+C{\left[ln\left(R_{T}\right)\right]}^{3}$$

and obtained:

A=−0.025278919295275;
B=0.009144982694036;
C=−0.00013756788807.

**Figure 7:**
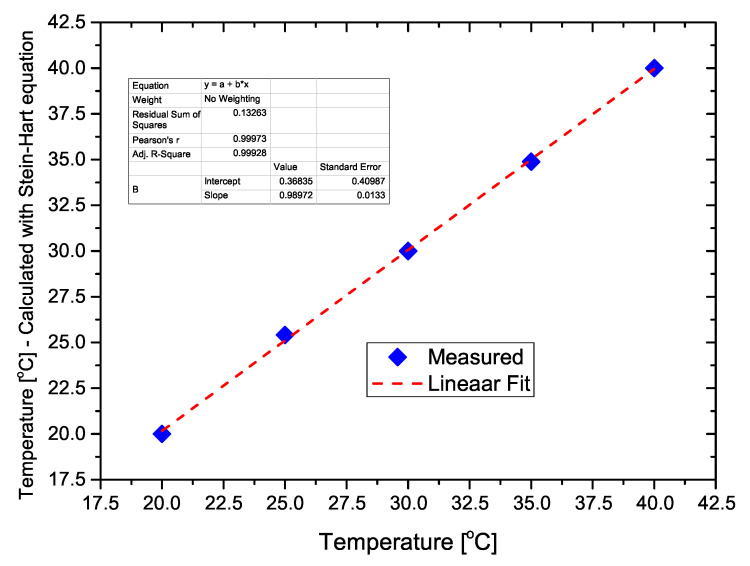
Calculated temperature using the Steinhart–Hart equation, as a function of the actual temperature.

A plot of the calculated temperature using the Steinhart–Hart equation as a function of the actual temperature is presented in Figure 7, showing that the developed thermoresistor follows the conventional thermistor equation.

### 5.3. Measuring Temperature with the ${\mathrm{SnSe}}_{2}$ Resistor

The response of the heat dissipation matric water potential sensor depends on the soil temperature [19], and it is important to be able to calibrate the sensor at different soil temperatures, to obtain a calibration curve [11]. Thus, it is important to check if we can use the value of $R_{T}$ when dissipating a very small amount of power (P≈2.8 mW), with 503 mV on it (what is obtained with PWM = 3.3%) to estimate the soil temperature.

**Figure 8:**
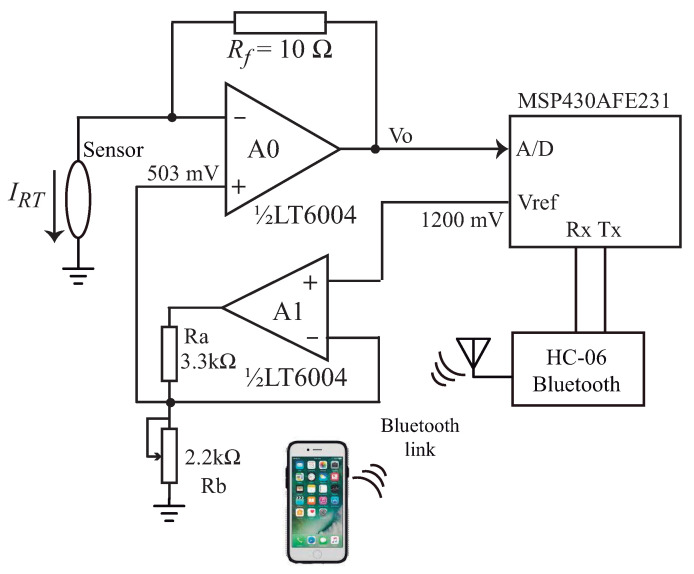
Circuit used to measure $R_{T}$, with V=503 mV applied on it, during 8 s.

In [14] it was shown that the soil temperature presented a very small variation ($T\approx 25\pm 2.5{\;}^{{}^{\circ}}$C), when measured during 205 days at a depth of 20 cm. The sensor was put in the thermal chamber and the value of $R_{T}$ was measured using the circuit of Figure 8, at three different temperatures ($T=20,\;25\;\mathrm{and}\;30{\;}^{{}^{\circ}}$C. This test is important to check if the self-heating of the thermosensitive resistor can be neglected when dissipating a very small amount of power (P≈2.8 mW) and, therefore, we can use the measured value of $R_{T}$ with 503 mV (PWM = 3.3%) on it to estimate the soil temperature.

In the set-up of Figure 8 the output of the MSP430AFE231 (Texas Instruments) microcontroller internal voltage reference $V_{REF}$ = 1200 mV is sent to a buffer (op-amp A1) and divided, in its output, with Ra−Rb, to obtain a voltage of 503 mV. This voltage is connected to the noninverting input of op-amp A0 (LT6004, from Analog Devices, Wilmington, MA, USA). The thermosensitive resistor is connected to the inverting input of A0, and the feedback provided by $R_{f}$ forces the voltage on $R_{T}$ to be 503 mV. Neglecting the input current of A0, the current that flows in $R_{T}$ comes from the output of A0 and passes through $R_{f}$, so we can write $V_{O}$ as:

(7) $$V_{O}=503\;\mathrm{mV}+R_{f}I\left(R_{T}\right)$$

Since

(8) $$R_{T}=\frac{503\;\mathrm{mV}}{I(R_{T})}$$

using Equations (8) and (9) we calculate the value of $R_{T}$ as:

(9) $$R_{T}=\frac{R_{f}}{\left(\frac{V_{O}}{503\;\mathrm{mV}}\right)-1}$$

The 503 mV is applied to $R_{T}$ during 8 s, and the voltage $V_{O}$ is measured with an internal 18 bits A/D converter (17 bit + 1 sign bit) of a MSP430AFE231 microcontroller (one conversion every 250 ms) and sent to a smartphone using a HC-6 bluetooth module. With 503 mV on it, the current in $R_{T}$ is approximately 5.6 mA, so the 5 pA input bias current of the LTC6004 can be neglected, as it was assumed when writing Equation (7).

**Figure 9:**
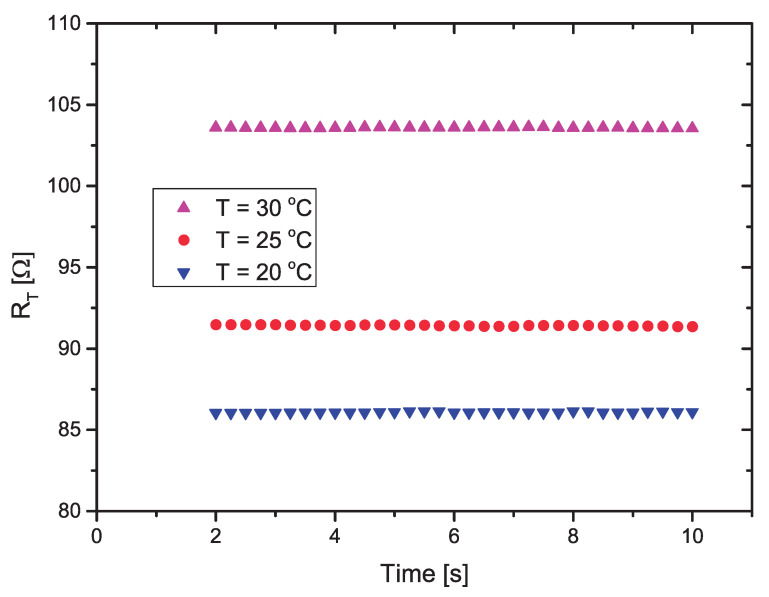
Measured values of $R_{T}$ with V=503 mV applied on it, as a function of the time, at three different temperatures.

The calculated values of $R_{T}$ for each temperature, using the measured values of $V_{O}$, are presented in Figure 9. As suggested in [11], to avoid transients in the response, after the power is applied, the first two seconds of measurement is discarded (eight measured points). Table 1 shows the mean values of $R_{T}$ ($\bar{R_{T}}$) and the standard deviation (σ), calculated from 32 points measured during 8 s (from 2 to 10 s). The results are plotted in Figure 9.

Thus, from the results presented in Table 1, in the 20–30 ${}^{{}^{\circ}}$C temperature range we notice that the standard deviation for all temperatures is almost four orders of magnitude smaller than the mean value, when we apply 503 mV to $R_{T}$ during 8 s. Therefore, the self-heating of $R_{T}$ can be neglected, and we can use its value to measure the soil temperature, using Equation (6) in the microcontroller.

### 5.4. Variation of the Power Dissipated in $R_{T}$

One important test was to observe how the power (and the resistance) changes if a constant voltage is applied on it, without any control circuit. We set PWM = 33.6% in the firmware, what results in a constant voltage $V_{sensor}=5$ V on $R_{T}$. The voltage on $R_{6}$ ($V_{R_{6}}$) was measured with the A/D converter (one conversion every 250 ms). For each conversion, both the current $I(R_{T})$ and the value of $R_{T}$ are calculated. Firstly $I(R_{T})$ is calculated using Equation (3); next, with $I(R_{T})$ known, since $V_{sensor}=5$ V, $R_{T}$ is calculated as:

(10) $$R_{T}=\frac{5V}{I(R_{T})}$$

**Figure 10:**
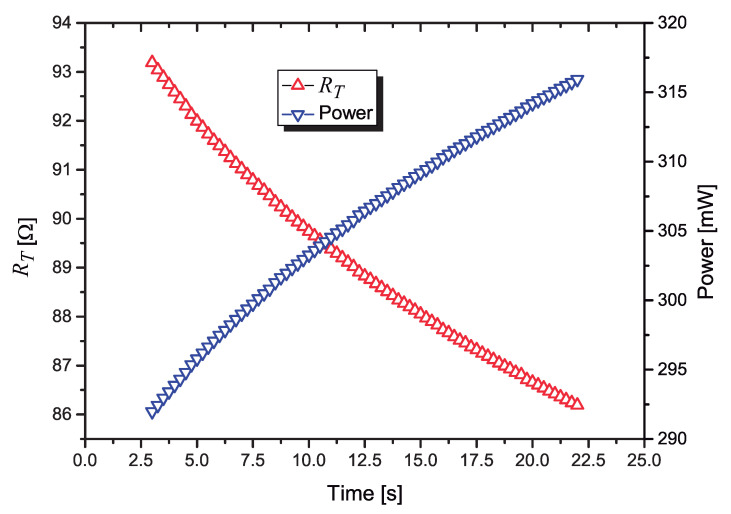
Variation of: $R_{T}$ (left axis); power dissipated in $R_{T}$ (right axis), a function of the time, when a constant voltage $V_{sensor}=5$ V is applied.

The values of $I(R_{T})$ and $R_{T}$ were transmitted, using the Bluetooth link, to a smartphone. The power dissipated in $R_{T}$ (P = $R_{T}\;I{\left(R_{T}\right)}^{2}$) and the values of $R_{T}$ are plotted in Figure 10.

### 5.5. Characterization of the Power Control Circuit

The prototype of the control circuit was built and several laboratory tests were performed. During the tests, as in the previous experiment, the A/D converter measures, at every 250 ms, the voltage $V_{R_{6}}$ (on $R_{6}=100\;\Omega$) and calculates $I(R_{T})$, given by Equation (3).

Using the measured values of $I(R_{T})$, the microcontroller calculates, for each conversion, the value of $R_{T}=V_{sensor}/I\left(R_{T}\right)$. These values of $I(R_{T})$ and $R_{T}$ are transmitted to a smartphone, via the Bluetooth link, along with other calculated parameters (the values of the PWM and $V_{sensor}$). Although we would need only to calculate and transmit the variation $\Delta R_{T}$, in this laboratory prototype all data is transmitted, so we can check if the circuit is operating correctly.

The firmware used in this version is as follows:

1. The routine starts with the control circuit turned-off and the circuit applying a very low voltage to the sensor during 2.5 s ($V_{sensor}=503$ mV, PWM = 3.3%), so that the power applied to the sensor is approximately 2.8 mW, to avoid the self-heating of $R_{T}$;
2. The first 1.75 s (seven measured points) are discarded, and the next three points (0.75 s) are measured and averaged to calculate the initial value of $R_{T}$. The microcontroller uses Equation (6) to calculate the soil temperature. This initial temperature is important to provide a temperature correction in the sensor calibration curves, as proposed in [19];
3. In our sensor, the target power in $R_{T}$ was set as P=325 mW. After the initial measurements at very low power, using the initial mean value of $R_{T}$, the microcontroller calculates the voltage $V_{sensor}$ that must be applied to $R_{T}$ to achieve P=325 mW as:
  (11) $$V_{sensor}=\sqrt{0.325\;R_{T}}$$
4. Next, using Equation (4), the new value of the pwm that generates this $V_{sensor}$ is calculated, set by the microcontroller and applied to the filter/op-amps;
5. $R_{T}$ starts to heat, and after 250 ms of self-heating an A/D conversion is performed and the new value of $I(R_{T})$ is calculated;
6. Using the last values of $V_{sensor}$ and $I(R_{T})$ the new value of $R_{T}$ is calculated;
7. With the current values of $R_{T}$ and $V_{sensor}$ the routine goes back to step #3 and repeats continuously for 20 s.

It is worth noting that the strategy used to control the power in $R_{T}$ is not based on the classical closed-loop control theory. Instead of calculating the error between the controlled process variable and the set-point and then applying a feedback, for each measured value of $R_{T}$ we calculate the exact required voltage that must be applied to $R_{T}$ to obtain the exact amount of power desired (P=325 mW) and set the PWM/filter/op-amps to furnish this voltage.

The first test performed was to check if the power applied to the sensor was constant, as desired for its proper operation. The test was performed with the sensor left in air (before it was inserted in the soil).

**Figure 11:**
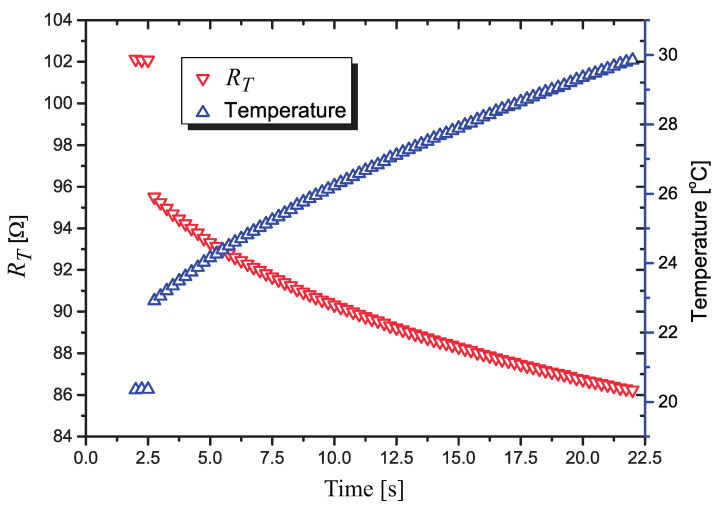
Variation of the value of $R_{T}$ and the temperature on it, when the signal processing circuit is used to control the power dissipated in $R_{T}$.

In the plot of in Figure 11 we present the calculated values of $R_{T}$ during a 22.5 s heat pulse. The voltages used to calculate $R_{T}$ are measured with a resolution of 18.3 μV by the 15 bit A/D converter. The first 1.75 s are discarded and the next 0.75 s (three points) are those calculated with P≈2.8 mW. The next points are measured with a nominal power of P=325 mW applied to $R_{T}$.

**Figure 12:**
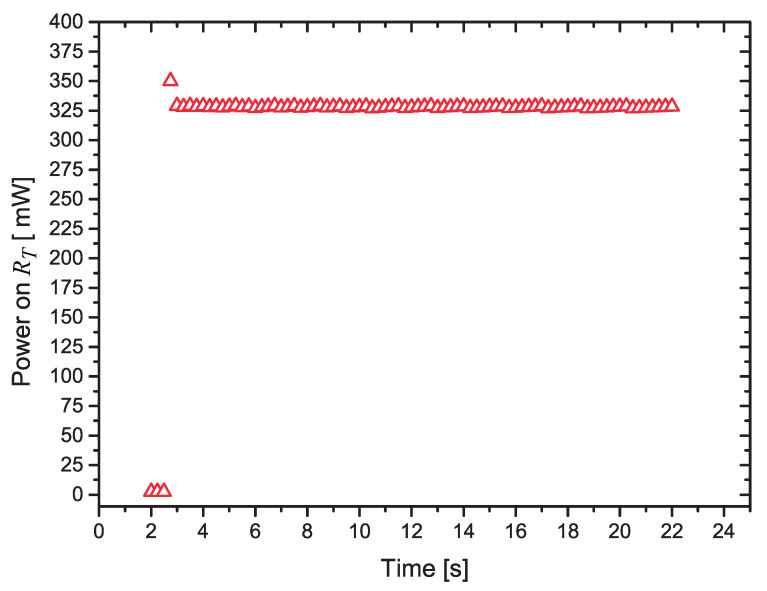
Variation of the power dissipated in $R_{T}$ as a function of the time.

In Figure 12 we present the calculated values of the power dissipated in $R_{T}$ during the 22 s heat pulse, the last 20 s with the control algorithm running.

We can notice that, after a small transient period (the first four points measured with the power at P=325 mW), the circuit can control the power very effectively. As suggested in [11], the first measurements after the power pulse is applied are discarded, to avoid transients in the measurement of the heater’s current, as it quickly ramps up. We discarded the first second (four measured points) after the heat pulse is applied.

Discarding only one measurement point after the power is applied, the calculated mean value of the power between 3 s and 23 s is $\bar{P}=327.98$ mW, with a standard deviation σ=0.59 mW.

**Figure 13:**
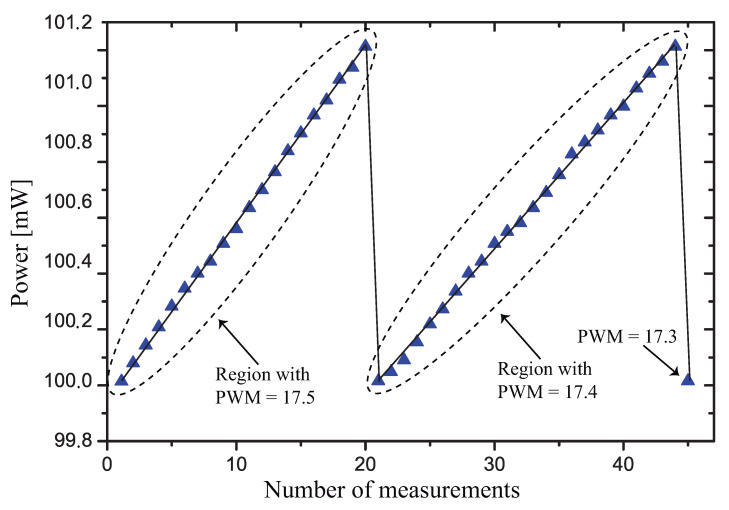
Variation of the power dissipated in $R_{T}$ during two PWM cycles.

It is worth discussing why the measured average value is different from the target value, P=325 mW. Observing the plot of Figure 13, where a test with P=100 mW was made, we see that, due to the discrete nature of the variation of the PWM, the measured power changes between 100.0 mW and 101.15 mW, and the mean value of the dissipated power was around 100.5 mW. The measured variation is in close agreement with the ΔP value calculated using Equation (5).

We used this test with P=100 mW because the self-heating (and consequently the dissipated power) changes more slowly with time, and we are able to measure more points during each value of the PWM, showing the operation of the control strategy.

**Figure 14:**
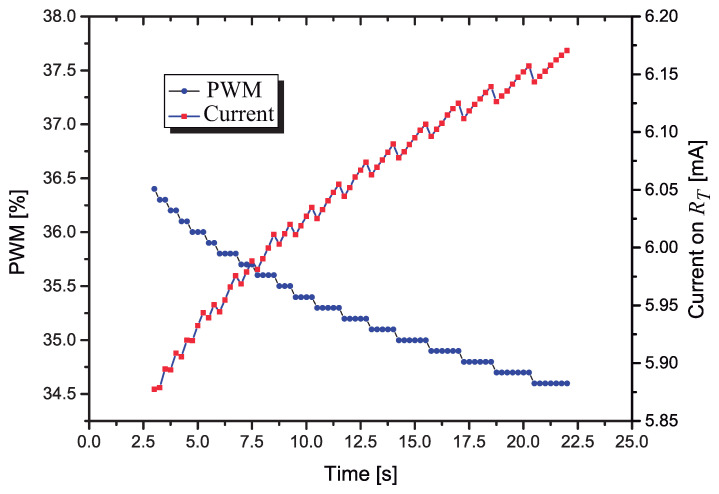
Variation of the PWM and $I(R_{T})$ as a function of the time, with the control circuit actuating.

In the plot of Figure 14 it is shown how the PWM and the current $I(R_{T})$ changes with respect to the time, when the control circuit is actuating. It is interesting to observe that the PWM decreases in fixed steps while the current on $R_{T}$ presents a saw-tooth behavior, increasing while the PWM is constant, with a fast decrease when a new (smaller) value of PWM is applied to $R_{T}$.

### 5.6. Characterization of the Sensor

Soil thermal conductivity depends both on the type of soil (loam, sand, silt, clay, etc.) and on the soil void ratio, amount of organic and mineral material. Therefore, soil water content sensors without porous blocks, which measure the thermal conductivity of the soil, need to be calibrated in the specific soil where they will be used.

Porous block matric water potential sensors, on the other hand, do not need to be characterized while inserted into the soil. The characterization can be performed in a laboratory bench, using, for example, a simple inverted Richards chamber [14,20]. After the calibration curve is obtained in laboratory, the soil water content of any type of soil can be obtained by consulting the soil water retention curve (WRC). A precise estimation of the WRC can be obtained using mathematical models, using the knowledge of the soil structure [21,22]. The use of porous blocks is so effective that it has been applied not only in heat dissipation sensors [11,23] but also in capacitive sensors, demonstrating its huge potential [24].

The thermosensitive resistor was encapsulated with a gypsum block with a radius of 50 mm and a height of 80 mm. No special attention was given to the preparation of the gypsum (porous control, porous uniformity, etc.) since our goal was only to test the technique of using a single thermosensitive resistor as heating and temperature measurement element.

In this work we measured the sensor inserted in the soil, so we can obtain the sensors sensitivity when the soil changes from dry to saturated. The following procedure was used to prepare the sensor:

1. Firstly, a PVC container with many small holes (about 1 cm of diameter) in the bottom was “dressed” with a pouch made of gauze, and the structure was weighted.
2. The container was filled with a loam clay soil, and put, with the sensor in a thermal chamber at $70{\;}^{{}^{\circ}}$C for 72 h;
3. After dried, both the container with soil and the sensor were weighted, and the sensor was inserted in the soil.
4. Next, a measurement of $R_{T}$ (soil dry) was taken using the developed circuit;
5. The container with the gauze in the bottom was put in a container that was slowly filled with water, and the soil was saturated by capillarity during 48 h.
6. After weighting the container, the soil gravimetric water content (mass of water/mass of soil) was calculated, $\theta_{w}=36.7$%.
7. Another measurement of $R_{T}$ (soil saturated) was taken.

To make sure that all the water had evaporated from the soil in the thermal chamber, after the experiment, the soil was transferred to a pyrex beaker, dried at a much higher temperature ($120{\;}^{{}^{\circ}}$C for 72 h) and weighted. The result showed that the soil was properly dried after its preparation in the thermal chamber.

**Figure 15:**
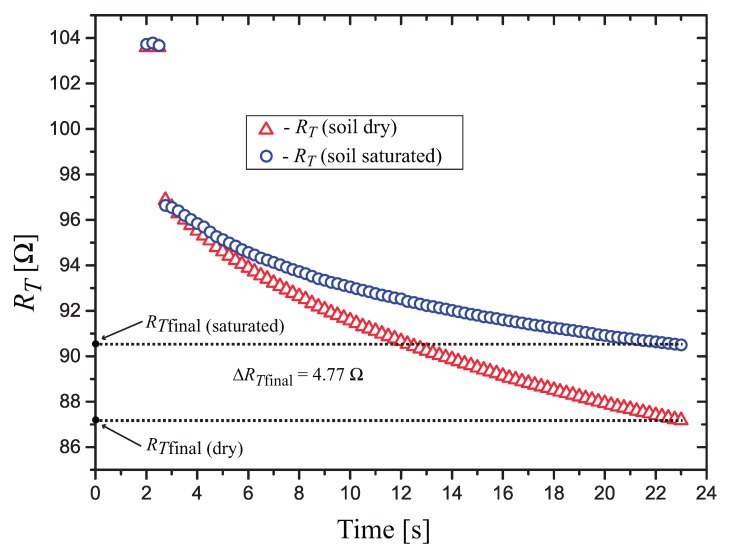
Measured values of $R_{T}$ for the sensor in the following soil conditions: dry and saturated ($\theta_{w}=36.7\%$).

The results of the measurements with the soil dry and saturated are shown in Figure 15. At the end of the heat pulse, we calculated an end-point difference in the resistance $\Delta (R_{Tfinal})=4.77\;\Omega$, a variation in $R_{Tfinal}$, from dry to saturated, of approximately 5.3%.

We obtained a 5.3% variation in $\Delta R_{Tfinal}$ in our sensor, while the sensor based on a bipolar transistor presented in [14] presented a variation of 1% in $\Delta V_{final}$. This difference in performance is due to the use of the high TC ${\mathrm{SnSe}}_{2}$ nanoparticle thermosensitive resistor. The presented solution (sensor and signal conditioning circuit) forms a complete low-cost matric water potential measurement system, suitable to be used in precision agriculture irrigation management.

## 6. Conclusions

A sensing system (sensor and signal processing circuit) for the measurement of soil matric water potential in precision agriculture irrigation management was presented. The sensor is based on the principle of the porous block sensor, where a porous material is allowed to equilibrate with the soil water. After an equilibrium is reached, the water content of the sensor/soil complex is measured and converted to a matric potential.

The sensor was encapsulated with a gypsum block, and when the soil water content changed from dry to saturated ($\theta_{w}=36.7\%$), we measured a variation of $\Delta R_{Tfinal}=4.77\;\Omega$ (corresponding to a temperature variation $\Delta T\approx 4.36{\;}^{{}^{\circ}}$C), and this results in an end-point sensitivity of S=130 mΩ/%. It is usually necessary to measure $\theta_{w}$ with a resolution of approximately $\Delta \theta_{w}=1\%$, and since the resistance is measured with a resolution three orders of magnitude smaller than the sensitivity S=130 mΩ/%, the developed system can easily meet this requirement.

Since the temperature rise ΔT observed in SHPP is typically only a few ${}^{{}^{\circ}}$C, to obtain a high resolution measurement of the $\Delta R_{T}$, a TC resistor is mandatory. A ${\mathrm{SnSe}}_{2}$ nanoparticles thermosensitive resistor was fabricated and presented a very high TC, from $-2.4\;\Omega {/}^{{}^{\circ}}$C) in the 20 to 25 ${}^{{}^{\circ}}$C to $-1.07\;\Omega {/}^{{}^{\circ}}$C in the 20 to 25 ${}^{{}^{\circ}}$C.

A low-cost signal processing circuit was developed to control the power and measure a single element heat dissipation soil water matric potential sensor based on thermosensitive resistor. The signal processing circuit, used to drive the sensor and maintain the power dissipated on the thermosensitive resistor constant, was based on a mixed signal circuit with simple analogue circuit and a MSP430AFE232 microcontroller. When a heat pulse was applied to the thermosensitive resistor during 20 s, the thermoresistor changed from 94.96Ω to 86.23Ω but the control circuit was able to control the dissipated power on it at P=327.98±0.3% mW.

## Figures and Tables

**Table 1:** Mean values of $R_{T}$ ($\bar{R_{T}}$) and standard deviation (σ), for *T* = 20, 25 and 30 ${}^{{}^{\circ}}$C.

	$T=20{\;}^{{}^{\circ}}$C	$T=25{\;}^{{}^{\circ}}$C	$T=30{\;}^{{}^{\circ}}$C
$\bar{R_{T}}\left(\Omega \right)$	103.58	91.43	86.08
σ	0.042	0.039	0.024

## Data Availability

Not applicable.
